# Phosphofructokinase 1 Platelet Isoform Promotes β-Catenin Transactivation for Tumor Development

**DOI:** 10.3389/fonc.2020.00211

**Published:** 2020-03-05

**Authors:** Jong-Ho Lee, Fei Shao, Jinjie Ling, Sean Lu, Rui Liu, Linyong Du, Jin Woong Chung, Sang Seok Koh, Sun-Hee Leem, Jichun Shao, Dongming Xing, Zhiqiang An, Zhimin Lu

**Affiliations:** ^1^Department of Biological Sciences, Dong-A University, Busan, South Korea; ^2^Cancer Institute of the Affiliated Hospital of Qingdao University, Qingdao Cancer Institute, Qingdao, China; ^3^Brain Tumor Center and Department of Neuro-Oncology, The University of Texas MD Anderson Cancer Center, Houston, TX, United States; ^4^State Key Laboratory of Oral Diseases, National Clinical Research Center for Oral Diseases, Chinese Academy of Medical Sciences Research Unit of Oral Carcinogenesis and Management, West China Hospital of Stomatology, Sichuan University, Chengdu, China; ^5^Key Laboratory of Laboratory Medicine, Ministry of Education of China, School of Laboratory Medicine and Life Science, Wenzhou Medical University, Wenzhou, China; ^6^Department of Urology, The Second Affiliated Hospital of Chengdu Medical College, China National Nuclear Corporation 416 Hospital, Chengdu, China; ^7^School of Life Sciences, Tsinghua University, Beijing, China; ^8^Texas Therapeutics Institute, Brown Foundation Institute of Molecular Medicine, University of Texas Health Science Center at Houston, Houston, TX, United States; ^9^Department of Hepatobiliary and Pancreatic Surgery and Zhejiang Provincial Key Laboratory of Pancreatic Disease of the First Affiliated Hospital, Institute of Translational Medicine, Zhejiang University School of Medicine, Hangzhou, China

**Keywords:** PFKP, β-catenin, c-Myc, cyclin D1, migration, invasion, proliferation

## Abstract

Metabolism plays a critical role in direct regulation of a variety of cellular activities via metabolic enzymes and metabolites. Here, we demonstrate that phosphofructokinase 1 platelet isoform (PFKP), which catalyzes a rate-limiting reaction in glycolysis, promotes EGFR activation-induced nuclear translocation and activation of β-catenin, thereby enhancing the expression of its downstream genes *CCND1* and *MYC* in human glioblastoma cells. Importantly, we showed that EGFR-phosphorylated PFKP Y64 has a critical role in AKT activation and AKT-mediated β-catenin S552 phosphorylation and subsequent β-catenin transactivation and promotion of tumor cell glycolysis, migration, invasion, proliferation, and brain tumor growth. These findings highlight a novel mechanism underlying a glycolytic enzyme-mediated β-catenin transactivation and underscore the integrated and reciprocal regulation of metabolism and gene expression, which are two fundamental biological processes in tumor development.

## Introduction

Increased transcriptional activity of β-catenin, which is essential for cell proliferation, migration, invasion, and survival (1, 2), has been detected in many types of human cancer (3–6). β-catenin transactivation leads to enhanced T-cell factor (TCF)/lymphoid enhancer factor (LEF)-driven transcription of genes, such as *CCND1* (encoding cyclin D1) and *MYC* (encoding c-Myc) (7–9). β-catenin can be activated not only by Wnt ligands but also by receptor tyrosine kinases, such as epidermal growth factor receptor (EGFR), whose mutation or overexpression of EGFR gene occurs in many types of human cancer, including more than 50% of glioblastoma (GBM) (10, 11). We previously showed that EGFR-induced β-catenin transactivation is regulated by mechanisms distinct from Wnt-dependent canonical signaling (12–14). EGFR activation-induced and CK2α-mediated α-catenin phosphorylation releases β-catenin from the β-catenin/α-catenin protein complex whereas nuclear pyruvate kinase M2 (PKM2) associates with β-catenin and induces gene expression by direct phosphorylation of histone H3 (12–17). In addition, AKT directly phosphorylates β-catenin at Ser552 (S552), which promotes nuclear translocation and transactivation of β-catenin (18).

Metabolic enzymes in cancer cells can possess non-metabolic functions and play critical roles in a variety in cellular functions (19–23). In the glycolytic pathway, phosphofructokinase 1 (PFK1), governing a rate-limiting step of glycolysis, catalyzes the conversion of fructose 6-phosphate and ATP to fructose-1,6-bisphosphate and ADP (24). PFK1 has PFK1 platelet (PFKP), PFK1 muscle (PFKM), and PFK1 liver (PFKL) isoforms, expressed differentially in different tissues and organs (24, 25). Our previous report showed that PFKP is the prominent PFK1 isoform in GBM cells and is overexpressed in human GBM specimens (26). Upon EGFR activation, K395-acetylated PFKP binds to EGFR, leading to EGFR-mediated phosphorylation of PFKP Y64, which in turn binds to an SH2 domain of p85 subunit of phosphoinositide 3-kinases (PI3K) and recruits PI3K to the plasma membrane. The activated PI3K and AKT enhances PFK1 activation and GLUT1 expression, thereby promoting aerobic glycolysis in cancer cells and brain tumorigenesis (27). However, the role of PFKP in the EGFR activation-induced β-catenin transactivation of GBM cells remains unknown.

In this study, we demonstrate that PFKP plays an instrumental role in EGFR activation-induced β-catenin transactivation in a PFKP Y64 phosphorylation-dependent manner, thereby regulating migration, invasion, and proliferation of GBM cells and brain tumor growth.

## Materials and Methods

### Materials

Mouse monoclonal antibodies for PFKM (sc-67028, 1:1,000 for immunoblotting), β-catenin (E-5, sc-7963, 1:200 for immunoblotting and 1:50 for immunofluorescence), and c-Myc (9E10, sc-40, 1:200 for immunoblotting) and polyclonal antibody for cyclin D1 (H-295, sc-753, 1:200 for immunoblotting) were purchased from Santa Cruz Biotechnology (Santa Cruz, CA). Rabbit polyclonal antibodies recognizing PFKP (12746, 1:1,000 for immunoblotting), PFKL (8175, 1:1,000 for immunoblotting), and β-catenin pS552 (9566, 1:1,000 for immunoblotting) were purchased from Cell Signaling Technology (Danvers, MA). Mouse monoclonal antibody for tubulin (clone B-5-1-2, T6074, 1:5,000 for immunoblotting) was purchased from Sigma (St. Louis, MO). Mouse monoclonal antibody for PCNA (610665, 1:1,000 for immunoblotting was purchased from BD Biosciences (San Jose, CA). Human recombinant EGF (01-407) was obtained from EMD Millipore (Billerica, MA). Hygromycin (400053), puromycin (540222), and G418 (345810) were purchased from EMD Biosciences (San Diego, CA). HyFect transfection reagents (E2650) were obtained from Denville Scientific (Metuchen, NJ). DAPI and Alexa Fluor 594 goat anti-mouse antibody were purchased from Molecular Probes (Eugene, OR).

### Cell Culture and Transfection

Non-small cell lung cancer A549 cells and GBM cells including U251, LN229, U87, and EGFRvIII-overexpressing U87 (U87/EGFRvIII) were maintained in Dulbecco's modified Eagle's medium (DMEM) supplemented with 10% bovine calf serum (HyClone, Logan, UT); these cells are routinely tested for mycoplasma. U87 and U251 cells were authenticated using short tandem repeat profiling at The University of Texas MD Anderson Cancer Center Characterized Cell Line Core Facility. Cells were plated at a density of 4 × 10^5^ per 60-mm dish or 1 × 10^5^ per well of a 6-well plate 18 h before transfection. Transfection was performed using HyFect transfection reagent (Denville Scientific) according to the manufacturer's instructions.

### DNA Constructs and Mutagenesis

Polymerase chain reaction (PCR)-amplified human PFKP was cloned into pcDNA3.1/hygro(+)-Flag vector. pLV/β-catenin deltaN90 (CA β-catenin) was purchased from Addgene (Cambridge, MA). pcDNA3.1/hygro(+)-Flag PFKP Y64F was created using the QuikChange site-directed mutagenesis kit (Stratagene, La Jolla, CA). shRNA-resistant (r) PFKP contained a448c, g450c, c453t, and c456g mutations.

The following pGIPZ shRNAs were used: control shRNA oligonucleotide, GCTTCTAACACCGGAGGTCTT; PFKP shRNA oligonucleotide, AGGAACGGCCAGATCGATA; PFKL shRNA oligonucleotide, AGTCTCTGAGATCTTACCT; PFKM shRNA oligonucleotide, TGATTTTCCCAGACATCCA.

### Quantitative Real-Time PCR Analysis

Total RNA isolation, reverse transcription (RT), and real-time PCR were conducted as described previously. The following primer pairs were used for quantitative real-time PCR: *CCND1*, 5′-TGCATGTTCGTGGCCTCTAA-3′ (forward) and 5′-TCGGTGTAGATGCACAGCTT-3′ (reverse); *MYC*, 5′-AAAGGCCCCCAAGGTAGTTA-3′ (forward) and 5′-GCACAAGAGTTCCGTAGCTG-3′ (reverse); *GLUT1*, 5′-CTGCTCATCAACCGCAAC-3′ (forward) and 5′-CTTCTTCTCCCGCATCATCT-3′ (reverse); *PKM2*, 5′-ATCGTCCTCACCAAGTCTGG-3′ (forward) and 5′-GAAGATGCCACGGTACAGGT-3′ (reverse); *LDHA*, 5′-TTGACCTACGTGGCTTGGAAG-3′ (forward) and 5′-GGTAACGGAATCGGGCTGAAT-3′ (reverse).

### Subcellular Fractionation

Nuclei were isolated using the Nuclear Extract Kit from Active Motif North America (Carlsbad, CA) and the ProteoExtract Subcellular Proteome Extraction Kit from Calbiochem (San Diego, CA), according to the manufacturers' instructions.

### Immunoblot Analysis

Extraction of proteins from cultured cells was performed using a lysis buffer (50 mM Tris-HCl, [pH 7.5], 0.1% SDS, 1% Triton X-100, 150 mM NaCl, 1 mM DTT, 0.5 mM EDTA, 100 μM PMSF, 100 μM leupeptin, 1 μM aprotinin, 100 μM sodium orthovanadate, 100 μM sodium pyrophosphate, and 1 mM sodium fluoride). Cell extracts were clarified via centrifugation at 13,400 *g*, and the supernatants (2 mg protein/ml) were subjected to immunoblot analysis with corresponding antibodies. The band intensity was quantified using Image Lab software program (Bio-Rad). Each experiment was repeated at least three times.

### Immunofluorescence Analysis

Cells were fixed and incubated with primary antibodies, Alexa Fluor dye-conjugated secondary antibodies, and DAPI according to standard protocols. Cells were examined using a deconvolution microscope (Zeiss, Thornwood, NY) with a 63-A° oil-immersion objective. Axio Vision software from Zeiss was used to deconvolute Z-series images.

### Luciferase Reporter Gene Assay

Transcriptional activities of TCF/LEF-1 in U87/EGFR and U87/EGFRvIII cells were measured as described previously. The relative levels of luciferase activity were normalized to the levels of untreated cells or to the levels of luciferase activity of the Renilla control plasmid. Each experiment was performed at least three times.

### Measurements of Glucose Consumption and Lactate Production

Cells were seeded in culture dishes, and the medium was changed after 6 h with non-serum DMEM. Cells were incubated for 24 h, and the culture medium was then collected for measurement of glucose and lactate concentrations. Glucose levels were determined by using a glucose (GO) assay kit (Sigma). Glucose consumption was the difference in glucose concentration between the collected culture medium and DMEM. Lactate levels were determined by using a lactate assay kit (Eton Bioscience, San Diego, CA). All results were normalized to the final cell number.

### Cell Migration and Invasion Assays

Cell migratory and invasive abilities were assessed by the transwell migration assay and Matrigel invasion assay (BD Biosciences, Franklin Lakes, NJ), respectively. Cells were trypsinized and were resuspended in serum-free DMEM. 1 × 10^5^ cells in 100 μL of serum-free medium were seeded in the upper chamber of transwell chambers (8-μm pore membranes; Corning). Membranes were coated with 0.78 mg/ml Matrigel for invasion assay. After 6 h (migration assay) or 24 h (invasion assay), cells on the upper surface of the filter were wiped off with cotton swabs. Cells on the lower surface of the filter were fixed with methanol and stained with hematoxylin and eosin. More than six views/insert were analyzed under a light microscope (200×), and stained cells were counted. Data represent mean ± standard deviation of three independent experiments.

### Cell Proliferation Assay

A total of 3 × 10^4^ cells were plated and counted at day 1, 3, 5, and 7 after seeding in DMEM with 0.5% bovine calf serum. Data represent the means ± SD of three independent experiments.

### Intracranial Implantation of GBM Cells in Mice

We injected 5 × 10^5^ U87/EGFRvIII GBM cells (in 5 μl of DMEM per mouse), with or without modulation of PFKP expression or CA β-catenin, intracranially into 4-week-old male athymic nude mice (seven mice/group), as described previously (27). The mice were euthanized 2 weeks after the GBM cells were injected. The brain of each mouse was harvested, fixed in 4% formaldehyde, and embedded in paraffin. Tumor formation and phenotype were determined by histological analysis of hematoxylin and eosin–stained sections. Tumor volume was calculated by 0.5 × L × W^2^ (L, length; W, width). All of the mice were housed in the MD Anderson Cancer Center (Houston, Texas) animal facility, and all experiments were performed in accordance with relevant institutional and national guidelines and regulations approved by the Institutional Animal Care and Use Committee at MD Anderson Cancer Center.

### Statistical Analysis

All quantitative data are presented as the mean ± SD of at least three independent experiments. A 2-group comparison was conducted using a 2-sided, 2-sample Student's *t*-test. A simultaneous comparison of more than two groups was conducted using 1-way ANOVA (SPSS statistical package, version 12; SPSS Inc.). Values of *P* < 0.05 were considered statistically significant.

## Results

### PFKP Expression Is Required for EGFR Activation-Induced Nuclear Translocation and Transactivation of β-Catenin

**Figure 1:**
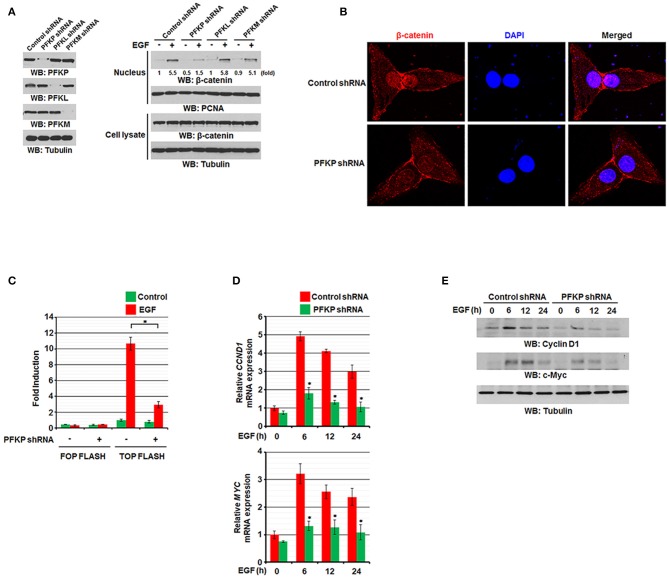
PFKP expression is required for EGFR activation-induced nuclear translocation and transactivation of β-catenin. **(A)** Serum-starved U87/EGFR cells with the indicated shRNAs (left panel) were treated with or without EGF (100 ng/ml) for 9 h. The cells were harvested for the isolation of nuclear fractions (right panel). Immunoblotting analyses were performed with the indicated antibodies. **(B)** U87/EGFRvIII cells were stably expressed with control shRNA or PFKP shRNA. Immunofluorescent staining was performed with an anti-β-catenin antibody. **(C)** U87/EGFR cells with or without PFKP depletion were transfected with TOP-FLASH or FOP-FLASH, which was followed by EGF treatment for 6 h. Luciferase activity was measured. The relative levels of luciferase activity were normalized to the levels of untreated cells and to the levels of luciferase activity in the Renilla control plasmid. Data represent the means ± SD of three independent experiments. ^*^P < 0.001, based on the Student's *t*-test. **(D,E)** Serum-starved U87/EGFR cells with or without depleted PFKP were treated with or without EGF for the indicated periods of time. The mRNA expression levels **(D)** and the protein expression levels **(E)** of *CCND1* and *MYC* in U87/EGFR cells were determined by real-time PCR and immunoblotting analyses with the indicated primers and antibodies, respectively. Data represent the means ± SD of three independent experiments. ^*^P < 0.001, based on the Student's *t*-test.

We previously reported that EGF stimulation results in translocation of β-catenin into the nucleus and increases its transactivation (11, 12). To examine the effect of PFKP expression on EGF-induced regulation of β-catenin in GBM cells, we used EGF to treat EGFR-overexpressing U87/EGFR and human GBM cells with or without expression of PFK1 short hairpin RNA (shRNA). Immunoblotting analysis of nuclear fractions of these cells showed that EGF-induced nuclear translocation of β-catenin was largely inhibited by depletion of PFKP, but not by depletion of PFKL or PFKM, whereas the total expression levels of β-catenin were not altered (Figure 1A). EGF-induced nuclear translocation of β-catenin was also largely inhibited by depletion of PFKP in LN229 GBM cells and A549 non-small cell lung cancer cells (Supplementary Figure S1A). Consistent with these findings, immunofluorescent studies revealed that depletion of PFKP in EGF-treated U87/EGFR cells (Supplementary Figure S1B) and U87 cells expressing the constitutively activated EGFRvIII mutant, which lacks 267 amino acids in its extracellular domain and is commonly found in GBM as well as in breast, ovarian, prostate, and lung carcinomas (28), resulted in decreased nucleus-localized β-catenin (Figure 1B). In addition, the TCF/LEF-1 luciferase reporter analysis showed that depletion of PFKP expression largely inhibited EGF-induced β-catenin transactivation in U87/EGFR, LN229, and A549 cells (Figure 1C and Supplementary Figure S1C). Moreover, immunoblotting and real-time PCR analyses showed that depletion of PFKP inhibited EGF-induced transcription of *CCND1* and *MYC* (Figure 1D and Supplementary Figure S1D) and their protein expression (Figure 1E and Supplementary Figure S1E) in U87/EGFR, LN229, and A549 cells. These results indicate that PFKP expression is required for EGFR activation-induced nuclear translocation and transactivation of β-catenin.

### PFKP Y64 Phosphorylation by EGFR Enhances AKT-Mediated β-Catenin S552 Phosphorylation and Nuclear Translocation of β-Catenin and Expression of Its Downstream Genes

**Figure 2:**
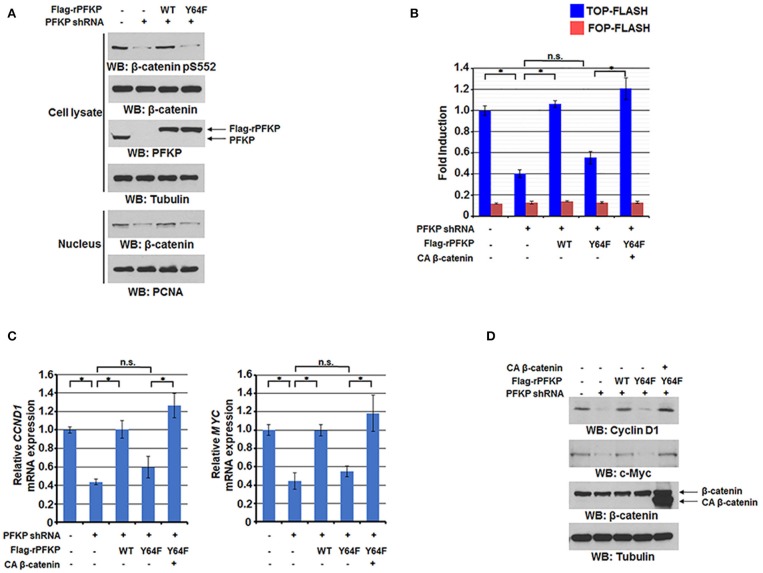
PFKP Y64 phosphorylation by EGFR enhances AKT-mediated β-catenin S552 phosphorylation and nuclear translocation of β-catenin and expression of its downstream genes. **(A)** Nuclear fractions of U87/EGFRvIII cells with or without PFKP depletion and with or without reconstituted expression of WT Flag-rPFKP or Flag-rPFKP Y64F mutant were prepared Immunoblotting analyses were performed with the indicated antibodies. **(B)** U87/EGFRvIII cells with or without PFKP depletion and with or without reconstituted expression of WT Flag-rPFKP or Flag-rPFKP Y64F mutant in the presence or absence of CA β-catenin expression were transfected with TOP-FLASH or FOP-FLASH. The relative levels of luciferase activity were normalized to the levels of luciferase activity of Renilla control plasmid. Data represent the means ± SD of three independent experiments. ^*^P < 0.001, based on the one-way ANOVA. **(C,D)** The mRNA expression levels **(C)** and the protein expression levels **(D)** of *CCND1* and *MYC* in U87/EGFRvIII cells with or without PFKP depletion and with or without reconstituted expression of WT Flag-rPFKP or Flag-rPFKP Y64F mutant in the presence or absence of CA β-catenin expression were determined by real-time PCR and immunoblotting analyses with the indicated primers and antibodies, respectively. Data represent the means ± SD of three independent experiments. ^*^P < 0.001, based on the one-way ANOVA.

We previously reported that EGFR-phosphorylated PFKP Y64 promotes PI3K activation (27). We next investigated the effect of PFKP Y64 phosphorylation on nuclear translocation and transactivation of β-catenin in response to EGFR activation. As expected, depletion of endogenous PFKP resulted in decreased AKT-mediated β-catenin S552 phosphorylation (Figure 2A), nuclear translocation (Figure 2A), and the TCF/LEF-1 transcriptional activity (Figure 2B). These decreases were rescued by reconstituted expression of RNAi-resistant (r) WT Flag-rPFKP, but not rPFKP Y64F mutant, in U87/EGFRvIII cells (Figures 2A,B). Consistent with these results, PFKP depletion-inhibited transcription of *CCND1* and *MYC* and their protein expression were rescued by reconstituted expression of WT Flag-rPFKP, but not rPFKP Y64F mutant, in U87/EGFRvIII cells (Figures 2C,D). Of note, the inhibitory effect of rPFKP Y64F expression on the transcriptional activity of TCF/LEF-1 (Figure 2B), the transcription of *CCND1* and *MYC* (Figure 2C), and their protein expression (Figure 2D) in U87/EGFRvIII cells was abrogated by expression of the constitutively active β-catenin (CA β-catenin) mutant (29). These results indicate that PFKP Y64 phosphorylation enhances EGFR activation-induced nuclear translocation and transactivation of β-catenin and expression of its downstream genes.

### PFKP Y64 Phosphorylation Promotes EGFR Activation-Enhanced GBM Cell Glycolysis, Proliferation, Migration, and Invasion

**Figure 3:**
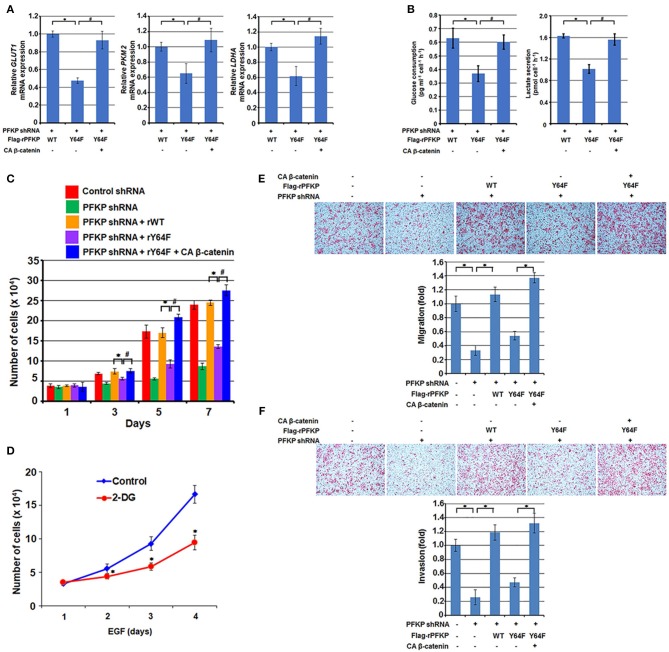
PFKP Y64 phosphorylation promotes EGFR activation-induced GBM cell glycolysis, proliferation, migration, and invasion. **(A,B)** Endogenous PFKP-depleted U87/EGFRvIII cells with reconstituted expression of WT Flag-rPFKP or Flag-rPFKP Y64F mutant in the presence or absence of CA β-catenin expression were cultured in no-serum DMEM for 24 h. The media were collected for analysis of glucose consumption **(A)** or lactate production **(B)**. All results were normalized to the final cell number. Data represent the means ± SD of three independent experiments. ^*^P < 0.001, ^#^*P* < 0.001, based on the one-way ANOVA. **(C)** U87/EGFRvIII cells with or without PFKP depletion and with or without reconstituted expression of WT Flag-rPFKP or Flag-rPFKP Y64F mutant in the presence or absence of CA β-catenin expression were cultured in 1% serum medium for the indicated periods of time and were harvested for cell counting. Data represent the means ± SD of three independent experiments. ^*^P < 0.001, ^#^*P* < 0.001, based on the one-way ANOVA. **(D)** U251 cells were pretreated with DMSO (Control) or 2-DG (10 mM) for 2 h and then the cells were treated with or without EGF (100 ng/ml) for the indicated periods of time. The cell numbers were counted. Data represent the means ± SD of three independent experiments. ^*^P < 0.001, based on the Student's *t*-test. **(E,F)** U87/EGFRvIII cells with or without PFKP depletion and with or without reconstituted expression of WT Flag-rPFKP or Flag-rPFKP Y64F mutant in the presence or absence of CA β-catenin expression were placed in serum-free medium in the upper transwell chamber or Matrigel-coated transwell chamber for migration **(E)** or invasion **(F)** assays, respectively. Six hours (migration assay) or twenty-four hours (invasion assay) after plating, cells that migrated to the opposite side of the insert were fixed with methanol and stained with hematoxylin and eosin. Representative microphotographs are shown (left panels). Stained cells were counted under a microscope (right panels). Data represent the means ± SD of three independent experiments. ^*^P < 0.001, based on the one-way ANOVA.

β-catenin transactivation is critical for EGFR activation-enhanced glycolysis, tumor cell proliferation, migration, and invasion for tumor development (11, 12, 16, 30). β-catenin transactivation enhances glycolysis through c-Myc-mediated upregulation of glucose transporter GLUT1, pyruvate kinase M2 isozyme (PKM2), and lactate dehydrogenase A (LDHA) expression (16). As expected, PFKP Y64F expression reduced mRNA expression of *GLUT1, PKM2*, and *LDHA* (Figure 3A), glucose uptake, and lactate production (Figure 3B) in U87/EGFRvIII cells, and this reduction was rescued by CA β-catenin expression in these cells (Figures 3A,B). PFKP Y64 phosphorylation enhances PI3K/AKT-dependent phosphofructokinase 2 (PFK2) phosphorylation and production of fructose-2,6-bisphosphate, which in turn promotes PFK1 activation (27), these results indicate that PFKP Y64 phosphorylation promotes glycolysis through more than one input.

We next examined the role of β-catenin regulation by PFKP Y64 phosphorylation in the EGFR activation-induced tumor cell proliferation. As shown in Figure 3C, depletion of PFKP largely inhibited tumor cell proliferation in U87/EGFRvIII cells, and this inhibition was alleviated by reconstituted expression of WT Flag-rPFKP, but not by rPFKP Y64F mutant. Of note, the defect in cell proliferation in U87/EGFRvIII cells expressing rPFKP Y64F was abrogated by expression of the active CA β-catenin mutant. Given that the AKT/β-catenin/c-Myc signaling cascade regulates not only glycolytic genes but also many other genes that contribute to cell proliferation, we examined the effect of inhibition of glycolysis on cell proliferation. As expected, treatment U251 cells with glycolysis inhibitor 2-deoxy-D-glucose (2-DG), which did not reduce EGF-enhanced AKT phosphorylation or c-Myc expression (Supplementary Figure S2), inhibited U251 cell proliferation (Figure 3D). These results suggest that PFKP Y64 phosphorylation-enhanced AKT phosphorylation and β-catenin transactivation induces a broad range of cellular effects that promotes cell proliferation.

In line with this finding, depletion of PFKP resulted in decreased tumor cell migration and invasion in a transwell migration assay (Figure 3E) and a Matrigel invasion assay (Figure 3F); this decrease was abrogated by expression of WT rPFKP, but not rPFKP Y64F (Figures 3E,F). As expected, the defect in cell migration (Figure 3D) and cell invasion (Figure 3F) in U87/EGFRvIII cells expressing rPFKP Y64F was rescued by expression of the CA β-catenin mutant (Figures 3E,F), indicating an essential role of PFKP Y64 phosphorylation-regulated β-catenin transactivation in EGFR activation-induced cell migration and invasion. Given that we measured cell migration at 12 h and cell invasion at 24 h, during which cell proliferation was not altered significantly among the cell lines (Figure 3C), the differences in cell migration and invasion were likely not caused by the differences in cell proliferation rates. These results indicate that PFKP Y64 phosphorylation promotes EGFR activation-enhanced tumor cell glycolysis, proliferation, migration and, invasion in a β-catenin transactivation-dependent manner.

### PFKP Y64 Phosphorylation-Induced β-Catenin Transactivation Promotes Brain Tumor Growth

**Figure 4:**
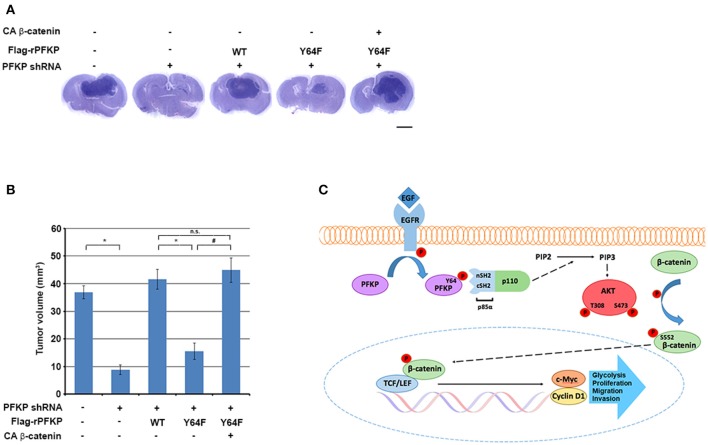
PFKP Y64 phosphorylation-induced β-catenin transactivation promotes brain tumor growth. **(A,B)** A total of 5 × 10^5^ PFKP-depleted U87/EGFRvIII cells with reconstituted expression of the indicated proteins was intracranially injected into athymic nude mice. After 2 weeks, the mice were euthanized and examined for tumor growth. Hematoxylin-and-eosin–stained coronal brain sections show representative tumor xenografts **(A)**. Tumor volumes were measured **(B)**. Data represent the means ± SD of seven mice (bottom panel). ^*^P < 0.001, ^#^*P* < 0.001, based on the one-way ANOVA; n.s., not significant. Scale bar, 2 mm. **(C)** EGFR-phosphorylated PFKP Y64 promotes AKT activation-mediated nuclear translocation of β-catenin, β-catenin transactivation, TCF/LEF-induced transcription of *CCND1* and *MYC*, leading to tumor development.

To determine the role of PFKP Y64 phosphorylation-induced β-catenin transactivation in brain tumorigenesis, we intracranially injected U87/EGFRvIII cells, U87/EGFRvIII–PFKP shRNA cells, and U87/EGFRvIII–PFKP shRNA cells with reconstituted expression of WT rPFKP or rPFKP Y64F mutant with or without CA β-catenin mutant into athymic nude mice. Depletion of PFKP significantly reduced the growth of brain tumors (Figures 4A,B), and this effect was abrogated by reconstituted expression of WT rPFKP, but not by that of rPFKP Y64F mutant (Figures 4A,B). Of note, the defect in the tumor growth U87/EGFRvIII cells expressing rPFKP Y64F was abrogated by expression of the active CA β-catenin mutant (Figures 4A,B). These results indicate that β-catenin transactivation induced by PFKP Y64 phosphorylation promotes brain tumor development.

## Discussion

Metabolism plays a critical role in the direct regulation of a variety of cellular activities via metabolic enzymes and metabolites, and deregulation of these metabolic activities has been implicated in human diseases, including cancer (20, 22). PFKP is a glycolytic enzyme that catalyzes the rate-limiting step of glycolysis by conversion of fructose 6-phosphate and ATP to fructose-1,6-bisphosphate and ADP. PFKP is overexpressed in human GBM specimens (26) and is required for EGFR activation-induced activation of PI3K and AKT (27). Here, we demonstrated that PFKP Y64 phosphorylation, which is mediated by EGFR, is required for EGFR activation-induced and AKT activation-dependent nuclear translocation of β-catenin, β-catenin transactivation, and TCF/LEF-induced transcription of *CCND1* and *MYC* and their protein expression. Importantly, PFKP Y64 phosphorylation promotes EGFR activation-induced GBM cell glycolysis, proliferation, migration, invasion, and brain tumorigenesis in a β-catenin transactivation-dependent manner (Figure 4C). These findings highlight the potential to target Y64-phosphorylation of PFKP for GBM treatment.

β-catenin transactivation in response to Wnt ligands and receptor tyrosine kinase activation has been detected in many types of human cancers (3–6). Notably, EGFR activation induces β-catenin transactivation through mechanisms distinct from canonical Wnt-induced signaling (12–14). PFKP functions as a rate-limiting metabolic enzyme in the regulation of glycolysis. We demonstrated that a small portion of PFKP translocates to the plasma membrane and acts as an instrumental signaling molecule in regulating PI3K/AKT activation upon EGFR activation (27). The critical role of PFKP in β-catenin transactivation and expression of its downstream gene targets to enhance aerobic glycolysis (27) and tumor cell growth and invasion underscores the integrated and reciprocal regulation of metabolism and gene expression, two fundamental biological processes in tumor development.

## Conclusions

PFKP promotes EGFR activation-induced nuclear translocation and activation of β-catenin in a PFKP Y64 phosphorylation-dependent manner, thereby enhancing the expression of β-catenin downstream genes *CCND1* and *MYC*. Thus, PFKP Y64 phosphorylation enhances tumor cell migration, invasion, proliferation, and brain tumor growth. These findings highlight the potential to target Y64-phosphorylation of PFKP for GBM treatment.

## Data Availability Statement

The datasets generated for this study are available on request to the corresponding author.

## Ethics Statement

The animal study was reviewed and approved by the Institutional Animal Care and Use Committee at MD Anderson Cancer Center.

## Author Contributions

J-HL, RL, FS, JL, SL, LD, and JS contributed the *in vitro* experiments. J-HL, JC, SK, and S-HL contributed the animal studies. J-HL, ZA, DX, and ZL wrote the manuscript.

### Conflict of Interest

The authors declare that the research was conducted in the absence of any commercial or financial relationships that could be construed as a potential conflict of interest.

## Supplementary Material

Supplementary Figure S1 PFKP expression is required for EGFR activation-induced nuclear translocation and transactivation of β-catenin. **(A)** Serum-starved LN229 and A549 cells with or without PFKP shRNA (left panel) were treated with or without EGF (100 ng/ml) for 9 h. The nuclear fractions were extracted (right panel). Immunoblotting analyses were performed with the indicated antibodies. **(B)** U87/EGFR cells expressing with control shRNA or PFKP shRNA were treated with or without EGF (100 ng/ml) for 9 h. Immunofluorescent staining was performed with an anti-β-catenin antibody. DAPI was used for staining DNA. **(C)** LN229 and A549 cells with or without PFKP depletion were transfected with TOP-FLASH or FOP-FLASH and then treated with EGF for 6 h. Luciferase activity was measured. The relative levels of luciferase activity were normalized to the levels of untreated cells and to the levels of luciferase activity in the Renilla control plasmid. Data represent the means ± SD of three independent experiments. ^*^*P* < 0.001, based on the Student's *t*-test. **(D,E)** Serum-starved LN229 and A549 cells with or without depleted PFKP were treated with or without EGF for 12 h. The mRNA expression levels **(D)** and the protein expression levels **(E)** of *CCND1* and *MYC* in LN229 and A549 cells were determined by real-time PCR and immunoblotting analyses with the indicated antibodies, respectively. Data represent the means ± SD of three independent experiments. ^*^*P* < 0.001, based on the Student's *t*-test.

Supplementary Figure S2 Inhibition of glycolysis does not reduce EGF-induced AKT phosphorylation or c-Myc expression. U251 cells were pretreated with DMSO (Control) or 2-DG (10 mM) for 2 h and then the cells were treated with or without EGF (100 ng/ml) for the indicated periods of time. Immunoblotting analyses were performed with the indicated antibodies.
