# Supplementary figures and images for: Phosphofructokinase 1 Platelet Isoform Promotes β-Catenin Transactivation for Tumor Development
Source: Front Oncol. 2020 Mar 5;10:211. doi: 10.3389/fonc.2020.00211 (PMC7066116; doi:10.3389/fonc.2020.00211)

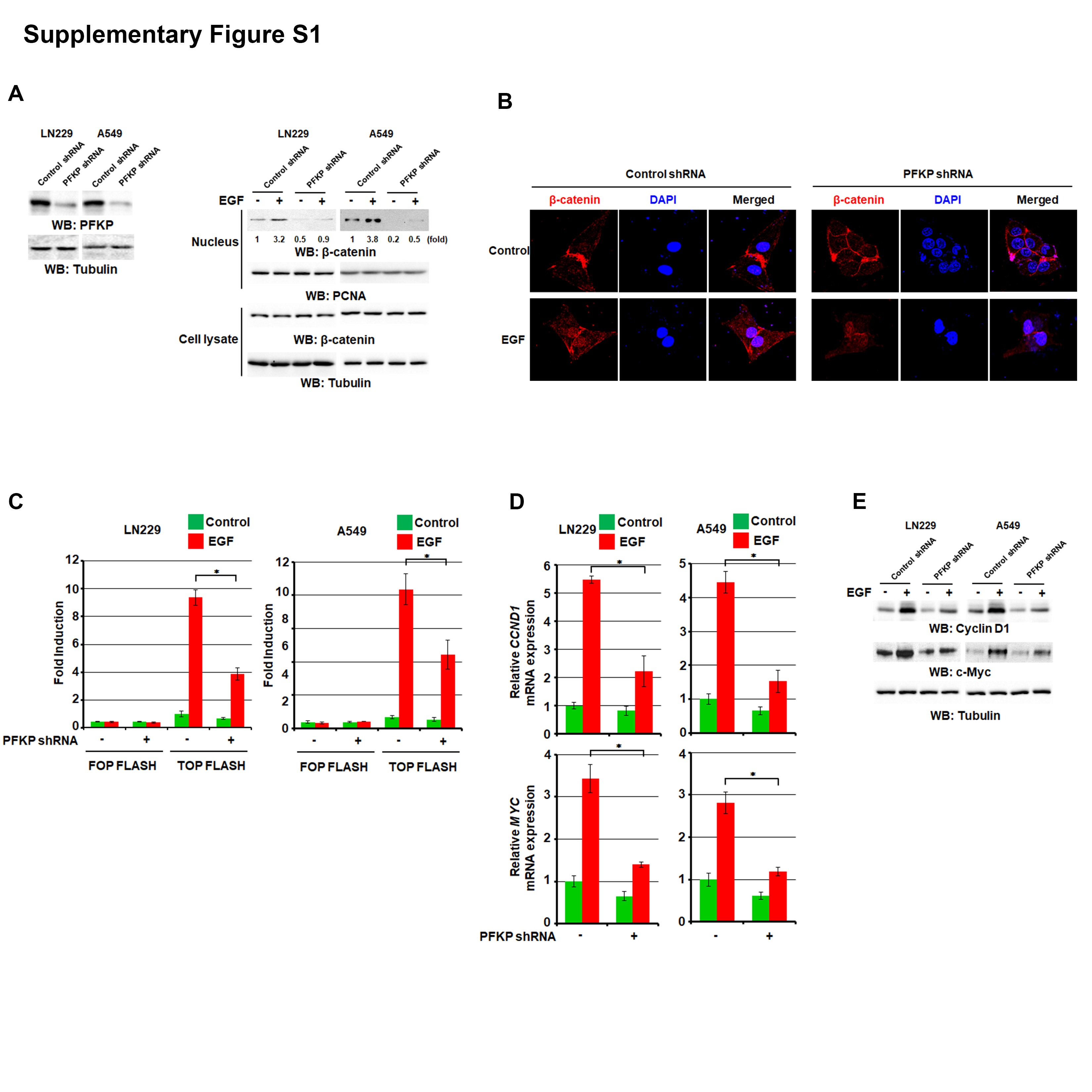

Supplement: Supplementary Figure S1 — PFKP expression is required for EGFR activation-induced nuclear translocation and transactivation of β-catenin. (A) Serum-starved LN229 and A549 cells with or without PFKP shRNA (left panel) were treated with or without EGF (100 ng/ml) for 9 h. The nuclear fractions were extracted (right panel). Immunoblotting analyses were performed with the indicated antibodies. (B) U87/EGFR cells expressing with control shRNA or PFKP shRNA were treated with or without EGF (100 ng/ml) for 9 h. Immunofluorescent staining was performed with an anti-β-catenin antibody. DAPI was used for staining DNA. (C) LN229 and A549 cells with or without PFKP depletion were transfected with TOP-FLASH or FOP-FLASH and then treated with EGF for 6 h. Luciferase activity was measured. The relative levels of luciferase activity were normalized to the levels of untreated cells and to the levels of luciferase activity in the Renilla control plasmid. Data represent the means ± SD of three independent experiments. *P < 0.001, based on the Student's t-test. (D,E) Serum-starved LN229 and A549 cells with or without depleted PFKP were treated with or without EGF for 12 h. The mRNA expression levels (D) and the protein expression levels (E) of CCND1 and MYC in LN229 and A549 cells were determined by real-time PCR and immunoblotting analyses with the indicated antibodies, respectively. Data represent the means ± SD of three independent experiments. *P < 0.001, based on the Student's t-test. [file Image_1.tif]

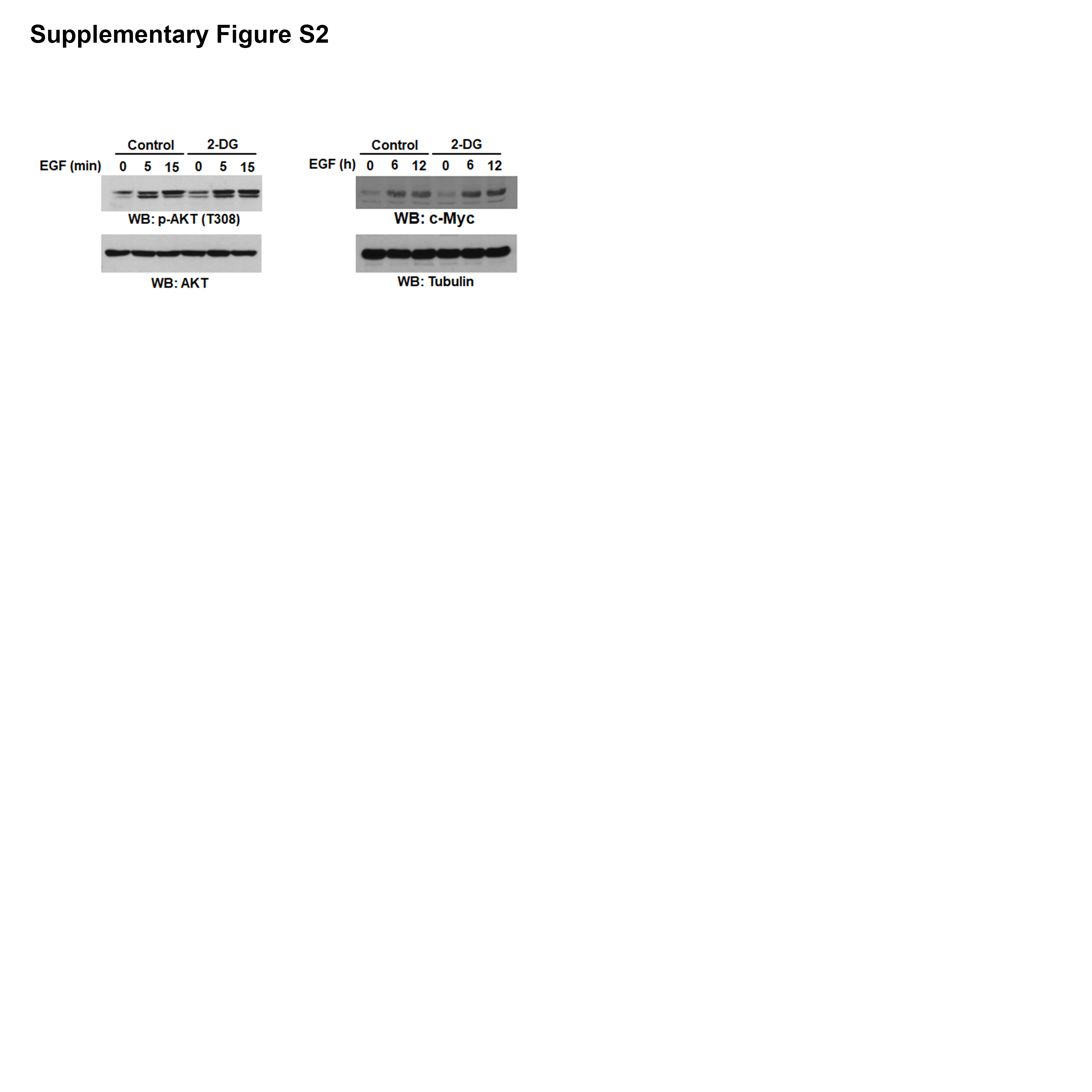

Supplement: Supplementary Figure S2 — Inhibition of glycolysis does not reduce EGF-induced AKT phosphorylation or c-Myc expression. U251 cells were pretreated with DMSO (Control) or 2-DG (10 mM) for 2 h and then the cells were treated with or without EGF (100 ng/ml) for the indicated periods of time. Immunoblotting analyses were performed with the indicated antibodies. [file Image_2.tif]
